# Macrophage-Related Testicular Inflammation in Individuals with Idiopathic Non-Obstructive Azoospermia: A Single-Cell Analysis

**DOI:** 10.3390/ijms24108819

**Published:** 2023-05-16

**Authors:** Peng Xia, Siwei Ouyang, Rong Shen, Zhao Guo, Guokun Zhang, Xiangwen Liu, Xuguang Yang, Kun Xie, Degui Wang

**Affiliations:** Department of Anatomy and Histology, School of Basic Medical Sciences, Lanzhou University, Lanzhou 730000, China

**Keywords:** bioinformatics, iNOA, infertility, macrophage, inflammation, spermatogenesis

## Abstract

Male infertility is a global issue that seriously affects reproductive health. This study aimed to understand the underlying causes of idiopathic non-obstructive azoospermia (iNOA), which is a type of male infertility with unknown origins that accounts for 10–15% of cases. By using single-cell analysis techniques, we aimed to uncover the mechanisms of iNOA and gain insight into the cellular and molecular changes in the testicular environment. In this study, we performed bioinformatics analysis using scRNA-seq and microarray data obtained from the GEO database. The analysis included techniques such as pseudotime analysis, cell–cell communication, and hdWGCNA. Our study showed a significant difference between the iNOA and the normal groups, indicating a disorder in the spermatogenic microenvironment in iNOA. We observed a reduction in the proportion of Sertoli cells and blocked germ cell differentiation. Additionally, we found evidence of testicular inflammation related to macrophages and identified *ODF2* and *CABYR* as potential biomarkers for iNOA.

## 1. Introduction

Infertility is a significant global health issue affecting a large percentage of couples worldwide, with male factors contributing to about 20–70% of cases [1]. Azoospermia, the absence of measurable sperm in the ejaculate, is a common condition associated with male infertility. Approximately 1% of the general male population and 10–15% of infertile men are affected by azoospermia, which represents a major impediment to successful reproduction [2,3].

Azoospermia is categorized into two main types: obstructive azoospermia (OA) and non-obstructive azoospermia (NOA). OA occurs when both testicles are blocked, leading to a lack of measurable sperm in the ejaculate. However, the spermatogenesis process remains normal in patients with OA. In contrast, NOA is not caused by blocked testicles, but rather by poor sperm production within the testes. There are currently three recognized phenotypes of NOA: Sertoli cell-only syndrome (SCOS), maturation arrest (MA), and hypospermatogenesis [4]. Azoospermia can result from various acquired causes, such as hypogonadism, CNS tumors, infection, anabolic steroid abuse, and others [1]. However, one study found that about 30–40% of patients had azoospermia, but no cause was found to explain why they were infertile [5]; these patients were defined as idiopathic non-obstructive azoospermia (iNOA). Therefore, clarifying the pathogenesis and dissecting potential biomarkers for iNOA is in urgent demand.

Some recent studies have identified significant differences between iNOA patients and normal individuals. The iNOA patients exhibit a distinct metabolic profile with hypergonadotropinemia and elevated levels of estradiol compared with normal individuals [6]. There were significant differences in Sertoli cells between iNOA patients and normal individuals, with evidence of Sertoli cell maturation arrest and activation of immune response-related pathways in iNOA [6]. A study conducted in dogs with iNOA also demonstrated chronic immune-mediated orchitis with increased immune cell infiltration [7]. Notably, some pathways, such as “phagocytosis”, suggested that a potential macrophage-related immunity within the testicular microenvironment in iNOA was activated [6,8].

The blood–testis barrier (BTB) is a crucial structure that is responsible for maintaining testicular immune privilege (TIP), which creates an environment that protects germ cells from attack by immune cells [9,10,11]. Recent reports have identified several cell types that regulate the testicular environment, including macrophages, dendritic cells, mast cells, and T cells [12,13].

Macrophages are involved in various processes, with their distinctive feature being participation in inflammation and immune regulation [14]. Generally, the macrophages can be divided into two subtypes, including the pro-inflammatory M1-type and the anti-inflammatory M2-type [15,16,17]. By maintaining a balance between pro-inflammatory and anti-inflammatory cytokines, testicular macrophages help to create a favorable environment for spermatogenesis and support the development of healthy sperm cells. Thus, testicular macrophages play a crucial role in regulating the testicular environment and maintaining the homeostasis of spermatogenesis in the testes.

However, if the balance of the testicular microenvironment is disturbed, the testicular macrophages present within the testes of infertile individuals can have a significant impact on the overall testicular environment [18]. For example, if there is a lack of Sertoli cells, resulting in damage to the blood–testis barrier (BTB), autoantigens may be exposed to the bloodstream [19]. This exposure can then activate macrophages, leading to damage to the entire testicular microenvironment [9]. Moreover, cytokines secreted during this process can also cause inflammation and further damage to the BTB, exacerbating testicular damage [20]. Testicular inflammation can lead to histopathological changes, such as thickening of the lamina propria, fibrosis, and granuloma-like changes [21]. These changes can furtherly damage the BTB and disrupt normal spermatogenesis, ultimately leading to spermatogenesis arrest [22].

Although macrophages play an important role in regulating the testicular environment of infertile individuals, their specific role in iNOA patients has not yet been clarified. Therefore, we hypothesized that macrophages may also play a role in the testicular environment of patients with iNOA. In recent years, breakthroughs in omics technologies, such as high-throughput sequencing and microarray technologies, have enabled us to explore and identify disease features in depth. Identifying the phenotypic characteristics and mechanisms of patients with iNOA using these technologies has significant clinical significance. In this study, we used bioinformatics technologies to investigate the immune environment in the testes and the role of testicular macrophages in iNOA. Our aim was to identify potential biomarkers and uncover the underlying causes of iNOA.

## 2. Results

**Figure 1:**
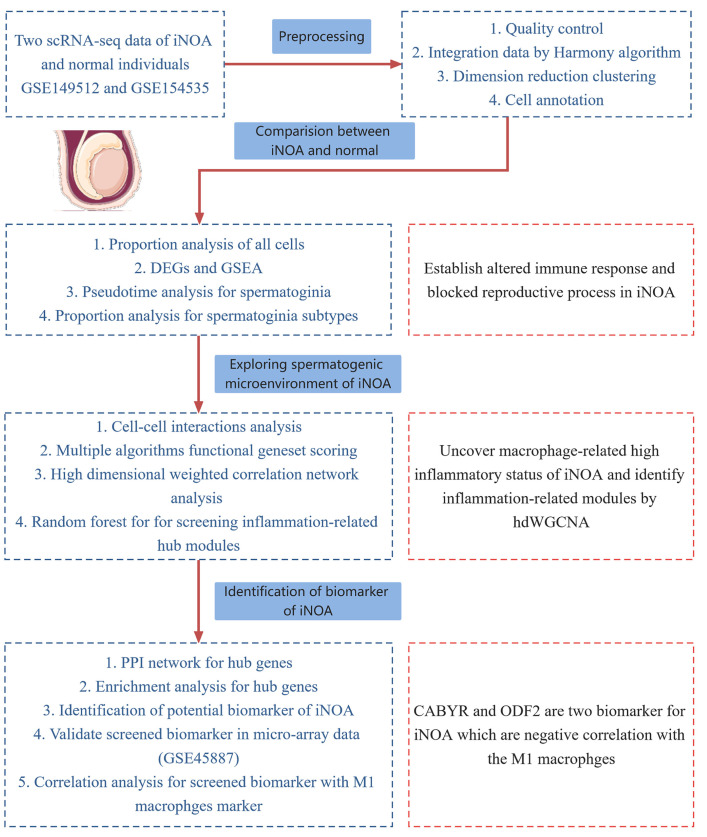
The flowchart of our study.

The study design workflow is depicted in Figure 1.

### 2.1. Single-Cell RNA Sequencing Data Quality Control and Preprocessing

**Figure 2:**
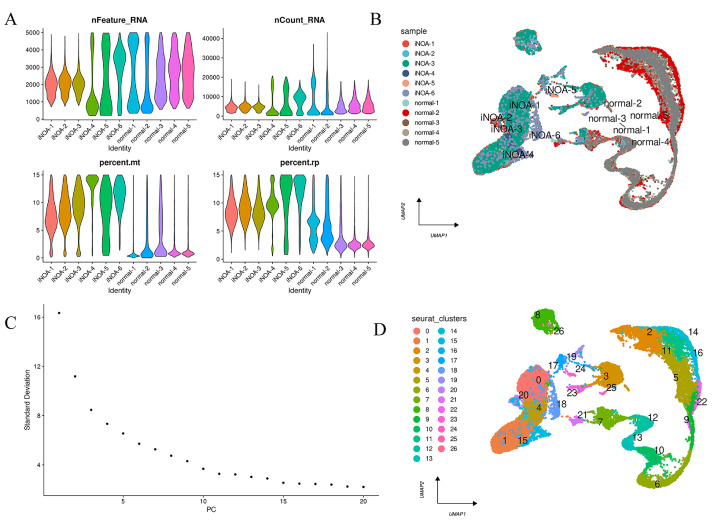
Preprocessing of scRNA-seq data for downstream analysis. (**A**) The features, counts, and percentages of mitochondrial genes and ribosome genes in each of the analyzed samples after quality control. (**B**) UMAP plot visualized the distribution of each sample after the integration of datasets using the harmony algorithm. (**C**) The first 15 principal components (PCs) were selected for further analysis. (**D**) A clustering algorithm with a resolution of 0.4 identified 26 clusters.

The quality control overview of our scRNA-seq data is presented in Figure 2A. Following preprocessing, we performed a harmony algorithm integration to effectively integrate and eliminate the batch effect. The landscape of the integrated 11 samples after the harmony algorithm is depicted in Figure 2B. The 1:15 principal components (PCs) and the harmony-based reduction were utilized for downstream analysis, as illustrated in Figure 2C. In accordance with the Seurat standard steps, we eventually identified 26 clusters, which were then embedded into the UMAP, as shown in Figure 2D. Ultimately, a novel combined dataset comprising 37,772 cells, including 5 normal samples and 6 iNOA samples, was utilized for further analysis.

### 2.2. The Annotation and Proportion Analysis of Cells

**Figure 3:**
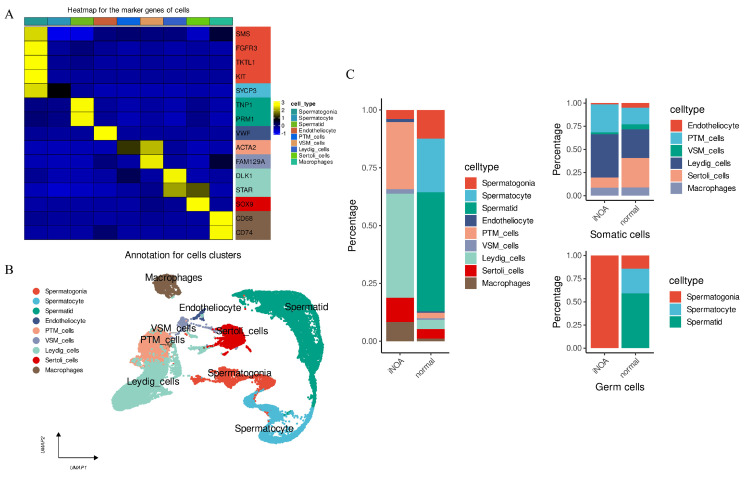
ScRNA-seq data were performed for annotation and to analyze the cell proportion distribution between iNOA and normal. (**A**) Heatmap to display the expression levels of marker genes in our identified cells. (**B**) UMAP plot shows the distribution of the nine cell types. (**C**) Cell fraction distribution differences between iNOA and normal. The figure presents three panels, with the left panel showing the overall cell fraction in the two groups being compared. The upper right panel represents the distribution of somatic cell fractions, while the lower right panel shows the distribution of germ cell fractions in the same groups.

A comprehensive evaluation was carried out to determine the cell type by merging pre-established cell markers with the CellMarker and PanglaoDB database. Supplementary Table S3 listed the annotation of each cluster. The marker genes of the defined cell types are presented in Figure 3A. Ultimately, the analysis verified nine cell types, all of which were utilized to generate a UMAP of their distribution (Figure 3B).

The distribution of cell types was analyzed in both iNOA and normal groups. A notable difference was observed, with the iNOA group having a lower proportion of germ cells compared with the normal group (Figure 3C, left). Further analysis divided the cells into germ and somatic cells. Somatic cells in the iNOA group showed a higher proportion of PTM and Leydig cells but lower levels of endotheliocytes, VSM cells, and Sertoli cells (Figure 3C, upper right). While macrophages were more prevalent in the iNOA group overall, there were no significant differences observed at the somatic cell level. This may be attributed to the decreased proportion of germ cells in iNOA, which amplified the somatic cell proportion overall. Germ cell analysis revealed that only spermatogonia were observed in iNOA, with no mature germ cells present (Figure 3C, lower right). This indicates that the iNOA group not only had a lower proportion of germ cells but was also absent of mature germ cells.

### 2.3. Altered Immune Response and Blocked Reproductive Processes in iNOA

**Figure 4:**
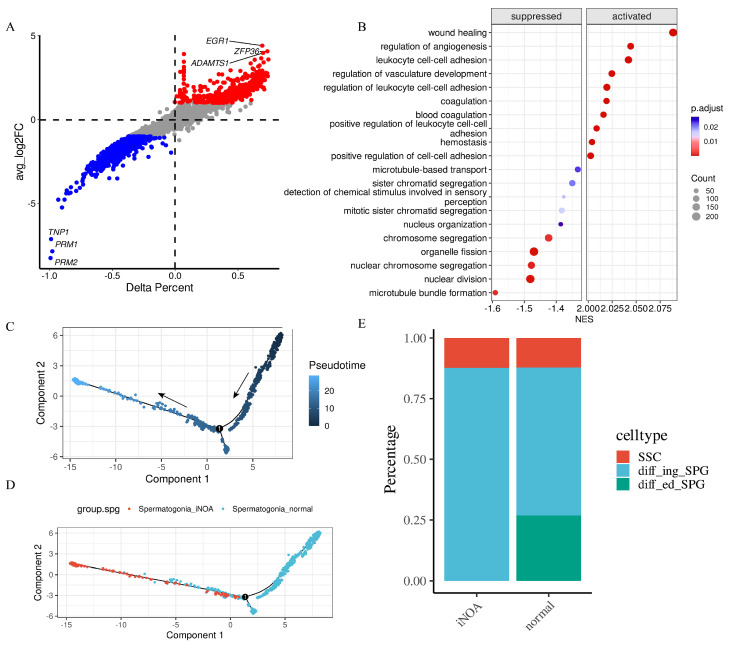
Significant difference was found between iNOA and normal. (**A**) Differential gene expression between iNOA and normal. (**B**) GSEA reveals that stress response pathways are activated and reproductive processes are suppressed in iNOA. (**C**) Trajectory analysis for spermatogonia displays the starting point in pseudotime with arrows. (**D**) Trajectory analysis reveals significant differences in spermatogonia between iNOA and normal. (**E**) Cell fraction analysis shows the differences of the three spermatogonia subtypes, including spermatogonia stem cells (SSC), differentiating spermatogonia (diff_ing_SPG), and differentiated spermatogonia (diff_ed_SPG).

The analysis of gene expression in iNOA cells compared with normal cells revealed a significant difference in the expression levels of many genes. We performed a differential gene expression analysis and presented the results in Supplementary Table S4. To better illustrate the differences in gene expression, we created Figure 4A, where the delta percent represented the percentage of a particular gene expressed in iNOA cells more or less than the background in normal cells. Our analysis showed that the top three upregulated genes in the iNOA group were *EGR1*, *ZFP36*, and *ADAMTS1*, whereas the top three downregulated genes were *PRM2*, *PRM1*, and *TNP1*, compared with the normal group.

We used gene set enrichment analysis (GSEA) to investigate the biological pathways and functional networks that were differentially impacted in iNOA cells compared with normal cells. Our results, presented in Figure 4B, revealed that stress response pathways were activated while reproductive processes were suppressed in the iNOA group. According to the results of GSEA, many stress pathways were activated in iNOA, which highlighted the presence of a stress response in the testes of iNOA patients. As iNOA is only diagnosed when no observable cause can explain the etiology, we believe that the activation of these stress response pathways was likely due to some internal reasons. In our study, we aimed to explore possible reasons to explain the condition of iNOA patients and we linked the stress response pathway with immune cells because immune factors have been identified as playing important roles in testicular stress. Altogether, this evidence provides support for the potential involvement of immune factors in males with iNOA and highlights the importance of further research to better understand their role in iNOA.

A pseudotime analysis was conducted on the spermatogonia of both the iNOA and the normal groups to investigate potential disparities between them. As shown in Figure 4C, the direction of the arrow indicates the start point in pseudotime. Analysis revealed a clear distinction between the two groups, as illustrated in Figure 4D. To further investigate these results, we subdivided the spermatogonia into three subtypes: spermatogonia stem cells (SSC), differentiating spermatogonia (diff_ing_SPG), and differentiated spermatogonia (diff_ed_SPG). Supplementary Table S5 and Supplementary Figure S1 provide more details on this process. The pseudotime analysis revealed significant differences between the spermatogonia of the iNOA and the normal groups. Specifically, we observed notable distribution differences in the pseudotime, which we believe were indicative of broader changes in the testicular environment. This change was consistent with our GSEA results, which manifested a suppressed reproduction process and an activated stress response in iNOA patients. We hold that the overall shift of spermatogonia in the testicular environment between the two groups results from the testicular stress response. These environmental changes are contributing to subsequent changes in the cells themselves.

We then performed a more detailed analysis of the proportion of each subtype, which revealed an absence of differentiated spermatogonia in the iNOA group, with no notable variation in the number of spermatogonia stem cells. As the differentiation of spermatogenic cells is a continuous progression, these findings suggest that there may be a block in the differentiation process in the iNOA group.

### 2.4. The iNOA Group Exhibited an Increase in Macrophage-Related Cell Interactions

**Figure 5:**
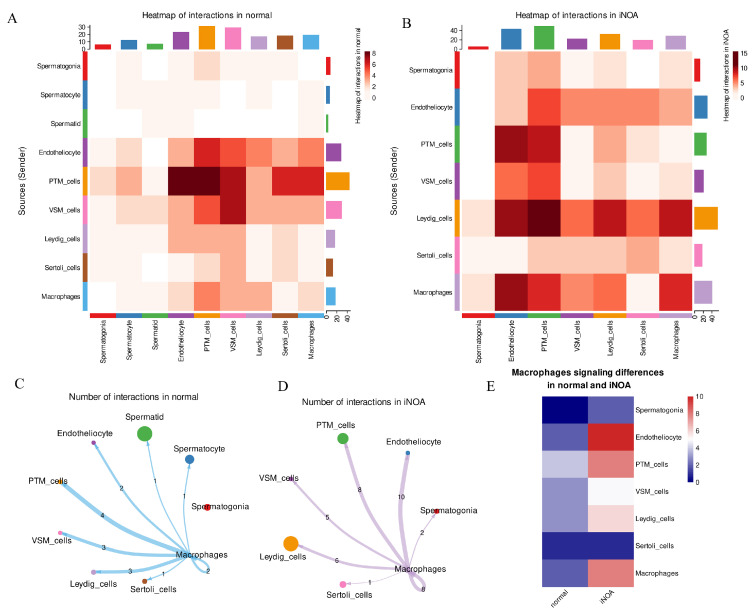
The analysis of cell–cell interactions showed an increase in macrophage signaling in iNOA. (**A**,**B**) Heatmap displays an overview of cell–cell interactions in both normal and iNOA groups. (**C**,**D**) Analysis of macrophages interacting with other cells in both iNOA and normal groups. (**E**) Heatmap demonstrates the differences in macrophage interactions between iNOA and normal groups.

We utilized the CellChat package to conduct a ligand–receptor pairs interaction analysis and to gain insight into the differences between iNOA and normal groups. Our analysis was performed on separate datasets for each group and revealed substantial variations in cell interactions in the iNOA group, specifically an increase in signaling originating from the macrophages. The interactions in the normal group are depicted in Figure 5A, while Figure 5B displays the interactions in the iNOA group.

The interactions between macrophages and other cells in iNOA and normal samples were analyzed and compared (Figure 5C,D). The results are presented in a heatmap (Figure 5E), which shows that, except for Sertoli cells, macrophages in iNOA tend to send more signaling to other cells, with endotheliocyte and VSM cells being the most affected. These findings suggest that macrophage-mediated signaling plays a crucial role in iNOA and impacts the somatic cells in the testes microenvironment.

Our previous GSEA analysis revealed an activated immune response in individuals with iNOA (Figure 4B). Based on the results of this ligand–receptor pairs interaction analysis, we furtherly revealed an increase in cell interactions signaling from the macrophages. Therefore, we have reason to believe that macrophages may play a major role in the wide-ranging immune activation observed in the testes of iNOA patients.

### 2.5. The Testes of the iNOA Group Showed Signs of Inflammatory Infiltration

To analyze the immune response in iNOA and normal groups, we used a functional gene set scoring method and evaluated the inflammatory signaling using gene signatures from MSigDB. We conducted three scoring algorithms (AddModuleScore, ssGSEA, and AUCell) and displayed the results at both overall and single-cell levels to demonstrate the differences between iNOA and normal groups. We also performed a Wilcoxon rank sum test for statistical analysis, with asterisks (*, **, ***, ****) indicating the level of significance (*p* < 0.05, *p* < 0.01, *p* < 0.005, *p* < 0.001, respectively).

**Figure 6:**
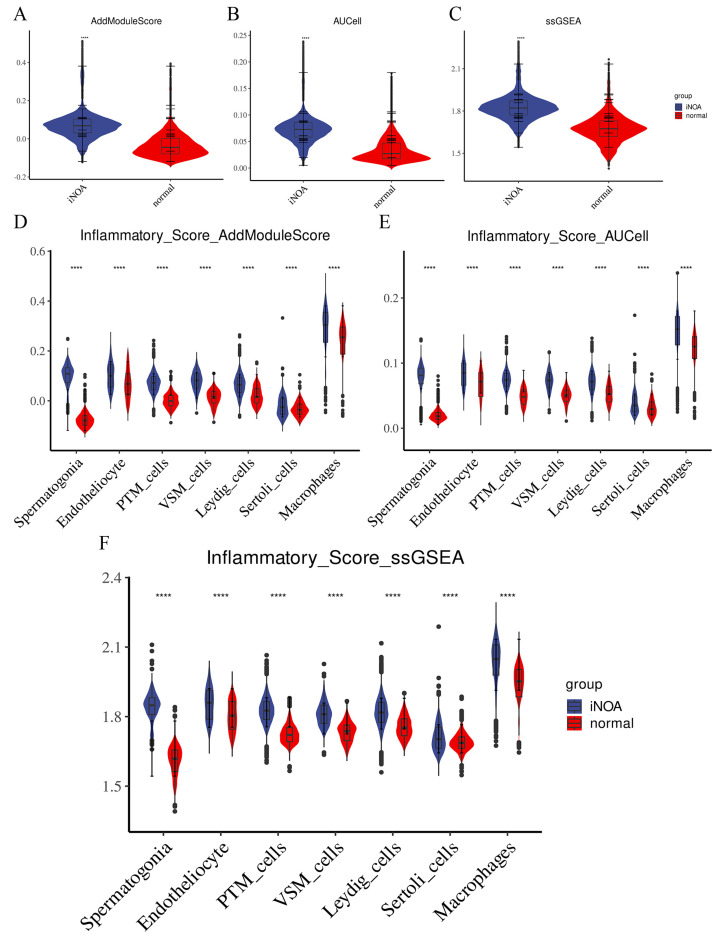
The testicular microenvironment in iNOA was characterized by a high level of inflammation. To evaluate the state of inflammation in iNOA and normal samples, we utilized three methods AddModuleScore, ssGSEA, and AUCell to calculate the inflammatory gene set score. We compared the overall inflammatory scores between iNOA and normal samples (**A**–**C**) and also evaluated the differences in inflammatory scores between iNOA and normal samples for each cell type (**D**–**F**). **** *p* < 0.001.

Our analysis revealed higher inflammatory scores in iNOA compared with normal overall (Figure 6A–C). Due to their absence in iNOA, spermatocyte and spermatid cells could not be analyzed. At the single-cell level, we also observed higher inflammatory scores in iNOA (Figure 6D–F). Interestingly, among all cells, macrophages displayed the highest inflammatory score, further bolstering the hypothesis that macrophage-related immune activation in iNOA may lead to an increase in inflammatory infiltration.

Overall, our results suggest that the testes of iNOA patients are subject to inflammatory infiltration, potentially caused by macrophage-related immune activation, which may be a key factor in iNOA-related infertility.

### 2.6. Screening for Inflammation-Associated Modules Using hdWGCNA

**Figure 7:**
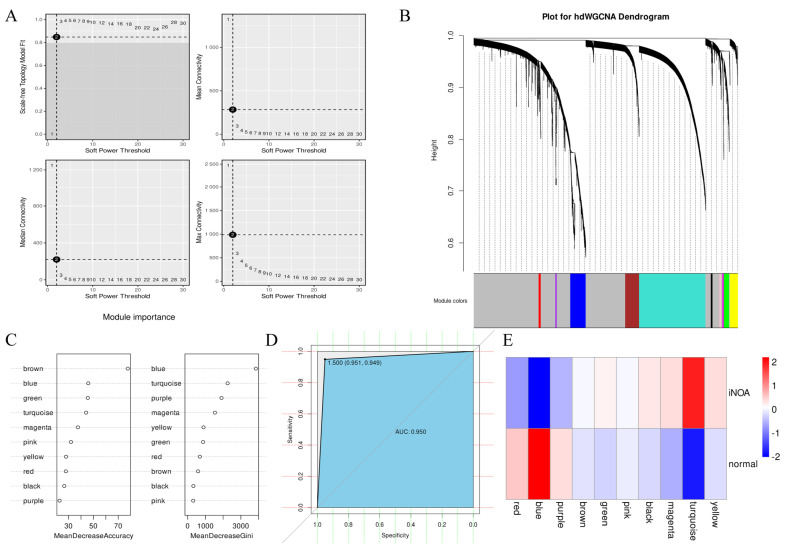
HdWGCNA revealed the blue and turquoise modules are two hub modules strongly associated with inflammation. (**A**) Soft power = 9 was selected to construct the scale-free network. (**B**) Dendrogram was utilized to visualize the 10 modules in the scale-free network. (**C**) Random forest algorithm was used to select inflammation-related modules by analyzing the module eigengenes (MEs). (**D**) Using the receiver operating characteristic curve (ROC) to evaluate the accuracy of our model. (**E**) Heatmap to compare the differences between iNOA and normal based on MEs.

To investigate the intrinsic properties of our single-cell RNA-seq data, a high-dimensional weighted correlation network analysis (hdWGCNA) was performed to decipher the gene expression differences between the iNOA and normal samples. A soft power value of 9 was selected to construct the co-expression network, as shown in Figure 7A. The final analysis resulted in ten modules, with the exception of the grey module, that were derived based on the scale-free network structure. The dendrogram for the modules is displayed in Figure 7B.

Module eigengenes (MEs) provide a holistic representation of gene expression patterns within each module and were utilized here to deduce the functional characteristics of the modules. The single-sample gene set enrichment analysis (ssGSEA) was previously executed to evaluate the gene expression levels and to determine the inflammation status of the iNOA and normal samples. Based on the ssGSEA results, the cells were divided into high and low inflammation groups via the median score. The combination of MEs and the high/low inflammation grouping information was then integrated as inputs for a random forest algorithm to identify modules that were related to inflammation. The algorithm was trained using 70% of randomly selected cells; the blue module was identified as the most influential one in terms of its contribution to differentiating the inflammation status, as shown in Figure 7C. The turquoise module was found to be the second most important, following the blue module. The remaining 30% of cells were used to evaluate the accuracy of the classification model; a favorable performance was demonstrated, as indicated by the receiver operating characteristic curve (ROC) of 0.950, as depicted in Figure 7D.

Next, we compared the MEs to investigate differences between the iNOA and the normal groups. A heatmap demonstrated that there was a large heterogeneousness in the pattern of gene expression in both groups (Figure 7E). Consistent with the previously screened inflammation-related modules, the blue and turquoise modules were found to be highly expressed in normal or iNOA samples and displayed significant expression differences between the two. In addition, a module correlation analysis was performed, which revealed a negative correlation between blue and turquoise (Supplementary Figure S2A). The turquoise module was highly expressed in iNOA. Through pathway enrichment analysis, multiple stress-related pathways were found enriched in turquoise (Supplementary Figure S2B). These findings indicated that a differential inflammation status was the most significant factor in distinguishing the iNOA and normal. In other words, our results suggest that high inflammation stress is a hallmark of iNOA, which provide valuable insights to a better understanding of the underlying mechanisms driving iNOA.

### 2.7. Identification of Potential Biomarkers for the Diagnosis and Monitoring of iNOA

**Figure 8:**
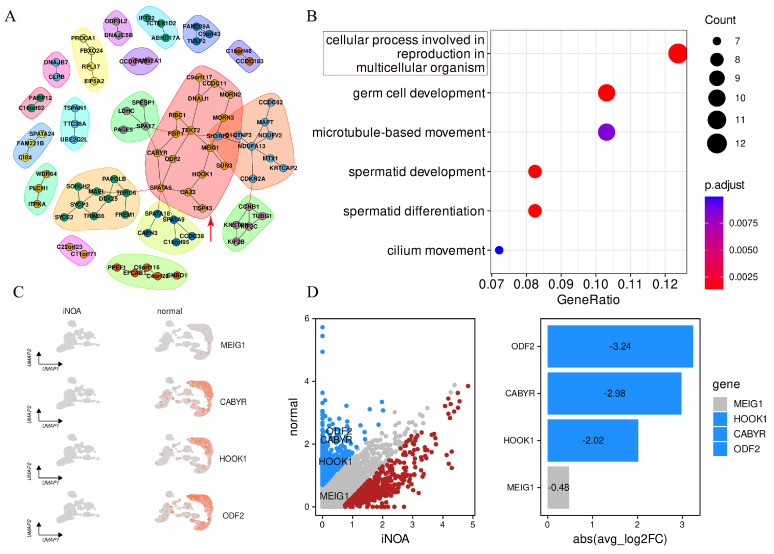
Screening for potential biomarkers of iNOA. (**A**) PPI network for the top 200 genes in the blue module. (**B**) GO enrichment analysis for the 200 genes to uncover their functional characteristics in reproduction. (**C**) UMAP plot to depict the expression patterns of the four intersecting hub genes (*MEIG1*, *CABYR*, *HOOK*, *ODF2*) in the iNOA and normal groups. (**D**) Expression differences of key genes in iNOA and normal (blue for downregulated and red for upregulated genes in iNOA).

To identify potential biomarkers for the diagnosis and monitoring of iNOA, we conducted a thorough analysis of the hub module selected from the hdWGCNA screening process. The expression of the blue module was strongly decreased in iNOA. Based on eigengene-based connectivity (kME), we identified the top 200 genes in the blue module to gain a comprehensive understanding of the functions of the genes within this module (Table S6). These genes were then subjected to further analysis through the STRING database to construct a protein–protein interaction (PPI) network. This network analysis allowed us to identify key hub genes that may play a crucial role in the regulation of gene expression in the iNOA group (Figure 8A).

In addition, the 200 genes were next performed in gene ontology (GO) enrichment analysis. The findings of the GO enrichment analysis, shown in Figure 8B, indicated a marked enrichment of genes related to reproduction processes. The most enriched pathway was “cellular process involved in reproduction in multicellular organism” (highlighted by the red rectangle). This pathway was further selected for intersection with the genes from the hub module in the protein–protein interaction (PPI) network (indicated by the red arrow). Our analysis ultimately led to the identification of four key genes: *MEIG1*, *CABYR*, *HOOK1*, and *ODF2*. The expression of these genes was furtherly mapped into the UMAP embedding to compare the differences between iNOA and normal, which showed an overall decreased expression in these genes in iNOA compared with the normal samples (Figure 8C).

To establish the significance of the differences in gene expression, we set a threshold of absolute log2 fold change (FC) > 1 and adjusted *p* value < 0.01. The blue/red points in Figure 8D represent genes that were significantly downregulated/upregulated in individuals with iNOA compared with the normal population, while the grey points indicate no significant difference between the two groups. In addition to *MEIG1*, which was only downregulated by −0.48, *ODF2* (−3.24), *CABYR* (−2.98), and *HOOK1*(−2.02) displayed significant downregulation in iNOA. These findings suggest that *ODF2*, *CABYR*, and *HOOK1* could serve as potential biomarkers for the diagnosis and monitoring of iNOA.

### 2.8. Examining the Correlation of ODF2 and CABYR with M1-Type Macrophages Markers

**Figure 9:**
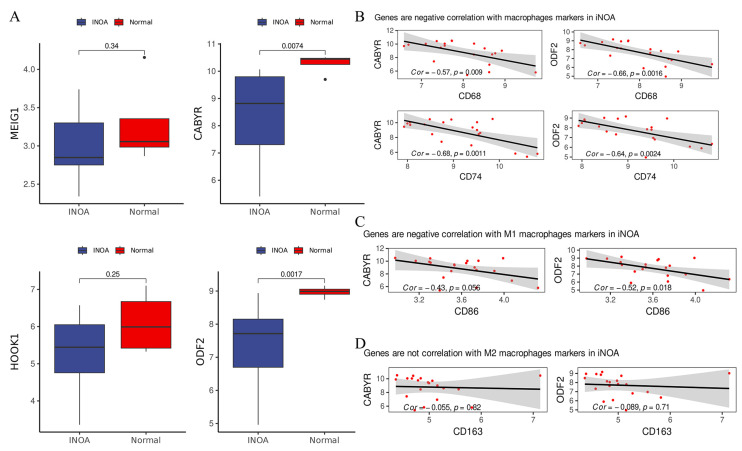
*CABYR* and *ODF2* are two biomarkers for iNOA which are negatively correlated with the M1 macrophages. (**A**) Compared the genes expression levels (*MEIG1*, *CABYR*, *HOOK*, and *ODF2*) in microarray data. (**B**) *CABYR* and *ODF2* had negative correlation with markers of macrophages (*CD68* and *CD74*). (**C**) *CABYR* and *ODF2* had negative correlation with markers of M1 macrophages (*CD86*). (**D**) *CABYR* and *ODF2* had no correlation with markers of M2 macrophages (*CD163*).

In order to corroborate our earlier findings, we analyzed a novel microarray dataset GSE45887 to examine the expression of four crucial genes (*MEIG1*, *CABYR*, *HOOK1*, and *ODF2*) in iNOA and normal groups. Our aim was to observe any disparity in the expression of these genes between the two groups. The results were consistent with our previous scRNA-seq data, as they indicated an overall decline in gene expression in iNOA patients (Figure 9A). Notably, *CABYR* and *ODF2* were considerably downregulated in iNOA, whereas *MEIG1* and *HOOK1* exhibited a less notable decrease in expression. Although the latter two genes also presented lower expression levels in iNOA, the difference turned out to be statistically insignificant. Our study therefore identified *CABYR* and *ODF2* as prospective biomarkers for iNOA.

Given the significant role of macrophages in increasing interactions with other cells and leading to heightened states of inflammation in iNOA, we investigated the potential relationship between macrophage-related immune responses and the expression of key genes, *CABYR* and *ODF2*, from the blue module that was closely associated with inflammation. We hypothesized that the decreased expression in *CABYR* and *ODF2* in iNOA may be a result of activated macrophages. We used *CD68* and *CD74* as two markers of macrophages and performed a correlation analysis with *CABYR* and *ODF2*. As shown in Figure 9B, a significant negative correlation was found, suggesting that the activation of macrophages may be a major contributing factor to the downregulation of *CABYR* and *ODF2* in iNOA.

High levels of inflammation were previously detected in iNOA and we recognized that the reduced expression in *CABYR* and *ODF2* was correlated with macrophage activation. Given the widely-accepted role of M1-type macrophages in upregulating inflammation, it is plausible that these macrophages could be accountable for the observed changes in gene expression in iNOA. As anticipated, our findings displayed a significant negative correlation between *CABYR* and *ODF2* and the marker for M1-type macrophages (*CD86*) (Figure 9C) but no significant correlation with the M2-type macrophage marker (*CD163*) (Figure 9D). These results reinforced the concept that inflammation in the testes of iNOA patients may be driven by pro-inflammatory macrophage activation. In summary, our analysis revealed a significant negative correlation between the expression of macrophage markers and key genes, which suggests that activated macrophages and ensuing inflammation may contribute to the downregulation of *CABYR* and *ODF2* in iNOA. These findings support the hypothesis that macrophage-related inflammation is involved in the pathogenesis of iNOA.

## 3. Discussion

It really is a complex process for spermatogenesis that is regulated by various factors, such as hormones and genes [23]. Our study has depicted an overall landscape for the testicular microenvironment of iNOA patients. A proper proportion of cells in the testicular microenvironment is essential for normal spermatogenesis. Our study has revealed a significant difference in cell proportion between iNOA and normal samples, with a notable decrease in germ cells and Sertoli cells in iNOA samples. In addition, macrophage-related high testicular inflammation in iNOA was also depicted. These findings enhanced our understanding of the etiology of iNOA, highlighting that testicular inflammation is a significant factor contributing to iNOA.

Testicular inflammation is a key factor in causing testicular damage in individuals with iNOA. In this study, we found a decreased Sertoli cell proportion in the testes of iNOA patients. The decreased proportion of Sertoli cells in iNOA patients may be linked to a disruption of the tight junctions (TJs) that make up the BTB [24]. The TJs are critical components of the BTB and play a vital role in protecting germ cells from the immune system and the lack of Sertoli cells indicates possible inflammatory-related damage to the BTB in iNOA patients. This damaging process is one of the main reasons for the decreased germ cell proportion and blocked germ cell differentiation observed in iNOA patients. We highlighted the importance of preserving the integrity of the BTB and the essential role played by Sertoli cells in maintaining its structure and function. Given this, we propose that anti-inflammatory drugs or other targeted therapies directed towards reducing testicular inflammation may be highly beneficial in treating iNOA patients. Anti-inflammatory treatments have been shown to restore fertility [24], which have an impact on restoring the BTB’s integrity and promoting spermatogenesis in iNOA patients [25].

We conducted hdWGCNA to gain critical insights into differential gene expression patterns between iNOA and normal groups. Subsequent module importance analysis highlighted the blue and turquoise modules as vital determinants for distinguishing iNOA and normal groups. Furthermore, functional enrichment analysis revealed that these two groups were enriched in reproduction and stress response processes, respectively. According to our analysis, the most changes in the testes of iNOA were the increased macrophage-related inflammation and the decreased spermatogenesis process in iNOA. Considering that the blue and turquoise modules corresponded to two distinct gene patterns with varying functional alterations, the former associated with decreased spermatogenesis and the latter with inflammation-related stress response, the gene expression of the blue module was particularly specific to the testis and could provide a more accurate means of assisting in the diagnosis of iNOA. Therefore, the blue module was chosen to screen potential biomarkers for iNOA. Eventually, we identified *CABYR* and *ODF2* from the blue module as two compelling biomarkers that displayed significant decreases in expression levels in iNOA patients.

*CABYR* (calcium binding tyrosine phosphorylation regulated)—a protein tasked with calcium activation and modulation—has been found to be downregulated in the sperm of iNOA patients [26], which has been demonstrated to inhibit flagellar function and compromise sperm motility [27]. Belonging to the outer dense fibers (ODFs) family, *ODF2* is an accessory cytoskeletal structure that safeguards the sperm tail from shear forces [28,29]. It has been reported that patients with *ODF2* deficiency and asthenozoospermia exhibit multiple abnormalities in sperm flagella morphology (MMAF) [30]. Based on our study, it seems that the decreased expression in the two genes is related to testicular inflammation and reduced spermatogenesis processes in iNOA patients, as the blue module had negative correlation with the turquoise. In addition, we found that the negative correlation between the expression of *ODF2* and *CABYR* with M1 macrophages provides compelling evidence that inflammation may play a key role in the pathogenesis of iNOA. We also compared various testicular inflammation markers, such as *IL-6*, *TNF-α*, *CXCL2*, *NLRP3*, and *CCL2*, to assess their expression differences between the iNOA and normal groups [31,32]. These genes are related to pro-inflammation and their upregulated expression levels indicate an inflammatory state. As shown in Supplementary Figure S3, consistent with our previous results, the expression levels of these genes were significantly higher in the iNOA group than in the normal group. These observations reinforced the link between high testicular inflammation mediated by macrophages and decreased spermatogenesis in iNOA patients. Furthermore, our study suggests that *ODF2* and *CABYR* may serve as promising biomarkers for the diagnosis of iNOA.

Overall, our findings highlight the potential impact of testicular inflammation on male infertility and emphasize the need for additional research into the mechanisms of inflammation in iNOA to develop effective treatment options. Anti-inflammatory agents may offer great promise for the treatment of iNOA patients, but further research is needed to investigate their efficacy and safety in treating inflammation-related male infertility. Our analysis indicates that the decreased expression in *CABYR* and *ODF2* may be linked to testicular inflammation and reduced spermatogenesis processes in iNOA patients. Collectively, our findings provide a valuable framework for further research into the molecular mechanisms underlying the development and progression of iNOA, as well as the identification of potential targets for therapeutic interventions.

There are still some limitations for this study. We only focused on some specific cell types (Sertoli cells, macrophages) to perform analysis that may ignore the global features to some extent; however, multiple cells are involved in the spermatogenesis process, such as Leydig cells. Although the Leydig cell proportion was observed as different between iNOA and normal, we did not conduct a further analysis. Additionally, our microarray data sample was not well-balanced, with only four normal samples and 16 iNOA samples, which may have introduced statistical bias when comparing the two groups. Finally, due to the lack of clinical samples, further studies are needed to validate our findings and to fully understand their underlying mechanisms

## 4. Materials and Methods

### 4.1. Datasets Acquisition

Our scRNA-seq data were obtained from the Gene Expression Omnibus (GEO) database [6,33], specifically from GSE149512 and GSE154535. GSE149512 contained 5 normal samples and 3 samples of iNOA, while GSE154535 contained 3 iNOA samples. A total of 11 samples were utilized in our analysis, including 5 normal and 6 iNOA samples. All samples used in our study were from adult individuals, with samples from GSE154535 aged 32, 37, and 41 years (GSM4673006, GSM4673007, and GSM4673008, respectively). However, we were unable to determine the age range for the samples in GSE149512, as this information was not provided by the original data source.

The microarray dataset GSE45887, which contained 4 normal samples and 16 samples of iNOA, was obtained from the GEO database [34]. This dataset was utilized as a means of verifying specific gene expression patterns in a multidimensional insight, in combination with the scRNA-seq data. 

### 4.2. Quality Control and Integration for ScRNA-Seq

The scRNA-seq data analysis was conducted using the Seurat (v4.3.0) (Seurat is an R package designed by the Satija lab for analyzing the single-cell RNA-seq data) in the R environment (v.4.0.2) [35]. Quality control measures were implemented by filtering out cells with mitochondrial genes > 15%, ribosome genes > 15%, and detected genes < 200 or >5000. The remaining cells underwent downstream analysis, which included normalization, selection of highly variable genes, scaling, and principal component analysis (PCA). To eliminate the batch effect of our combined scRNA-seq data, we utilized the harmony package in accordance with its official guidelines [36]. Following the harmony integration process, we performed uniform manifold approximation and projection (UMAP) via the RunUMAP function. Cell clusters were identified using the FindNeighbors and FindClusters (resolution = 0.4) functions. All standard analysis procedures were conducted using default parameters.

### 4.3. Cell Annotation

To ensure the accuracy of cell annotation, we utilized the FindAllMarkers function to identify differentially expressed genes (DEGs) in each cluster. We combined this information with online databases such as the CellMarker database (http://xteam.xbio.top/CellMarker/ (accessed on 1 January 2023)) [37] and PanglaoDB database (https://panglaodb.se/ (accessed on 1 January 2023)) [38]. Additionally, we used predefined markers from the published literature to make a comprehensive judgment for the ultimate cell type (Supplementary Table S1). Specifically, spermatogonia are represented by *SMS* [6], *FGFR3* [39], *TKTL1* [40], and *KIT* [41]; spermatocyte by *SYCP3* [42]; spermatid by *TNP1* [43] and *PRM1* [43]. Endotheliocytes are denoted by *VWF* [44], peritubular myoid cells by *ACTA2* [45], vascular smooth muscle cells by *FAM129A* [46], Leydig cells by *DLK1* [47] and *STAR* [47], Sertoli cells by *SOX9* [48], and macrophages by *CD68* [49] and *CD74* [49]. *CD86* denotes pro-inflammatory M1 macrophages, while *CD163* denotes anti-inflammatory M2 macrophages [50,51].

### 4.4. GSEA and Trajectory Analysis

To identify any differences between the iNOA and normal groups, we performed a gene expression analysis. The criteria for determining differential gene expression were based on a threshold of absolute log2 fold change (FC) > 1 and adjusted *p* value < 0.01. This threshold allowed for the selection of genes with significant differences in expression between the two groups.

After obtaining the differential gene expression analysis results, we sorted the average log_2_FC for gene set enrichment analysis (GSEA) using the clusterProfiler package [52,53]. The GSEA results provided a higher level of functional interpretation of the raw differential gene expression data and shed further light on the biological processes involved. The results were visualized using the dotplotGsea function.

To investigate the disparities in the evolution of spermatogonia between the iNOA and normal groups, we conducted a trajectory analysis utilizing the Monocle2 package [54]. This sophisticated tool enabled us to transform the genes into a reversed graph embedding and deduce the trajectory of our targeted cells by implementing a dimensionality reduction technique to arrange the cells in pseudotime. The plot_cell_trajectory function was employed to visualize the results.

### 4.5. Inference and Analysis of Cell–Cell Communication

To gain a comprehensive understanding of the cell–cell interactions, we utilized the CellChat package to infer and analyze the communication between cells [55,56]. By leveraging the ligand–receptor pairs in CellChatDB, we performed separate analyses of the cell–cell interactions in both the iNOA and the normal groups. Furthermore, we projected the gene expression data onto protein–protein interaction (PPI) networks to ensure the accuracy of the inferred outcomes.

### 4.6. Evaluating Inflammatory Score

We obtained a set of 200 genes related to the inflammatory response from the MSigDB (Molecular Signature Database) (Supplementary Table S2). To assess the gene-set enrichment score for each cell, we utilized three algorithms: AddModuleScore from the Seurat package, single-sample gene set enrichment analysis (ssGSEA) from the GSVA package, and score single cells with gene regulatory networks (AUCell) from the AUCell package. Visualization was performed using the ggplot2 R package [57].

### 4.7. High-Dimensional Weighted Correlation Network Analysis

The biological system is an intricately complex entity. The weighted gene co-expression network analysis (WGCNA) is a potent technique that deciphers specific gene expression patterns involved in multiple biological processes [58]. However, the conventional WGCNA is not applicable to the trait of scRNA-seq data. The high-dimensional weighted correlation network analysis (hdWGNCA) is a novel algorithm that provides a highly modular approach and can construct co-expression networks across multiscale cellular and spatial hierarchies, which is more suitable for scRNA-seq data [59].

Therefore, we employed hdWGNCA for the analysis of our scRNA-seq data. We selected genes that were expressed in at least 5% of cells to construct the hdWGNCA object, which was then transformed into a Metacells object. The co-expression network was constructed with a soft power of 9 for subsequent analysis. All standard downstream analyses were conducted according to the official pipeline, which can be found at https://smorabit.github.io/hdWGCNA/articles/basic_tutorial.html (accessed on 1 January 2023).

Module eigengenes (MEs) can reflect the gene expression profile of an entire co-expression module. Cells were classified as having either high- or low-inflammatory status based on the ssGSEA-evaluated median inflammatory score and randomly divided in a 7:3 proportion for further analysis. The MEs of each module in the selected 70% of cells were combined with the random forest algorithm to screen for inflammation-related modules. The random forest algorithm, based on the random forest package, utilized mean decrease accuracy and mean decrease Gini to reflect the importance of modules [60]. The remaining 30% of selected cells were used to test the accuracy of the random forest model using the receiver operating characteristic curve (ROC), based on the pROC package [61]. Hub genes, which were highly connected in modules, were calculated using the eigengene-based connectivity (kME).

### 4.8. Protein–Protein Interactions and Functional Enrichment Analysis

The hub genes in our screened important module underwent protein–protein interaction analysis in the STRING database [62] and the igraph package was utilized for visualization [63]. Functional enrichment analysis was performed using the clusterProfiler package and the Metascape website (please see https://metascape.org/gp/index.html#/main/step1 (accessed on 1 January 2023)) [52,64].

## 5. Conclusions

In conclusion, our study has expanded the testicular landscape of iNOA at the single-cell level, contributing to a broader understanding of this disorder. Our analysis revealed key features of the spermatogenic microenvironment, including a reduction in the proportion of Sertoli cells and blocked germ cell differentiation. We also provided evidence of macrophage-related testicular inflammation and identified *ODF2* and *CABYR* as potential biomarkers in iNOA. These findings have the potential to inform future research and clinical practice in this area and contribute to a more comprehensive understanding of the underlying mechanisms of iNOA.

## Data Availability

The data used to support the findings of this study are available from the corresponding authors upon request.

## Supplementary Materials

The supporting information can be downloaded at: https://www.mdpi.com/article/10.3390/ijms24108819/s1.
