# Supplementary material for: Macrophage-Related Testicular Inflammation in Individuals with Idiopathic Non-Obstructive Azoospermia: A Single-Cell Analysis
Source: Int J Mol Sci. 2023 May 16;24(10):8819. doi: 10.3390/ijms24108819 (PMC10218437; doi:10.3390/ijms24108819)
Supplement: Supplementary file 1 [file ijms-24-08819-s001.zip › Supplementary figures.docx]

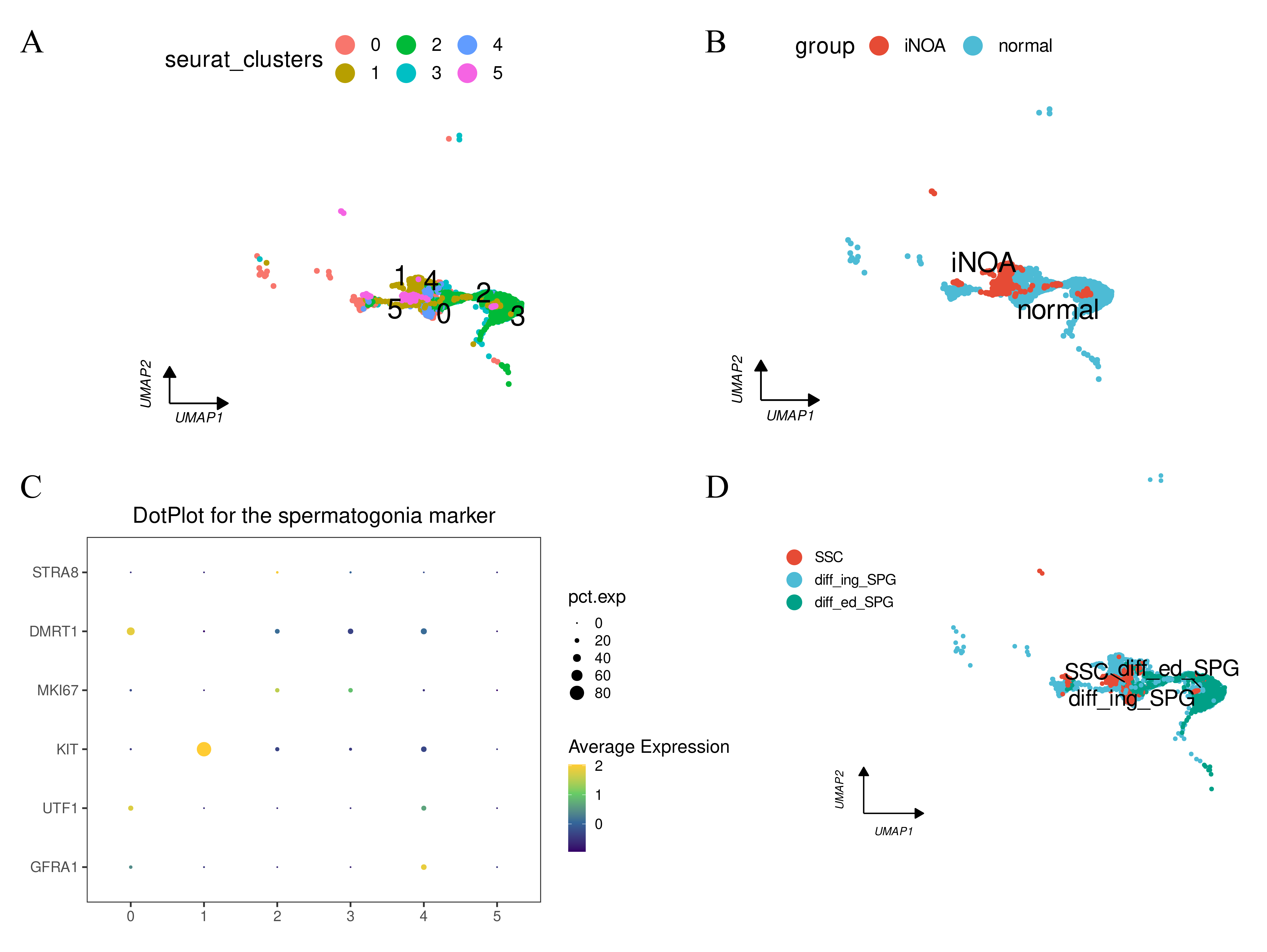


Figure S1: (A) 5 clusters were identified through clustering algorithm for spermatogonia. (B) UMAP plot for the spermatogonia distribution in iNOA and normal. (C) Dotplot for the marker genes of spermatogonia subtypes. (D) UMAP plot for the three spermatogonia subtypes, including spermatogonia stem cells (SSC), differentiating spermatogonia (diff_ing_SPG), and differentiated spermatogonia (diff_ed_SPG).


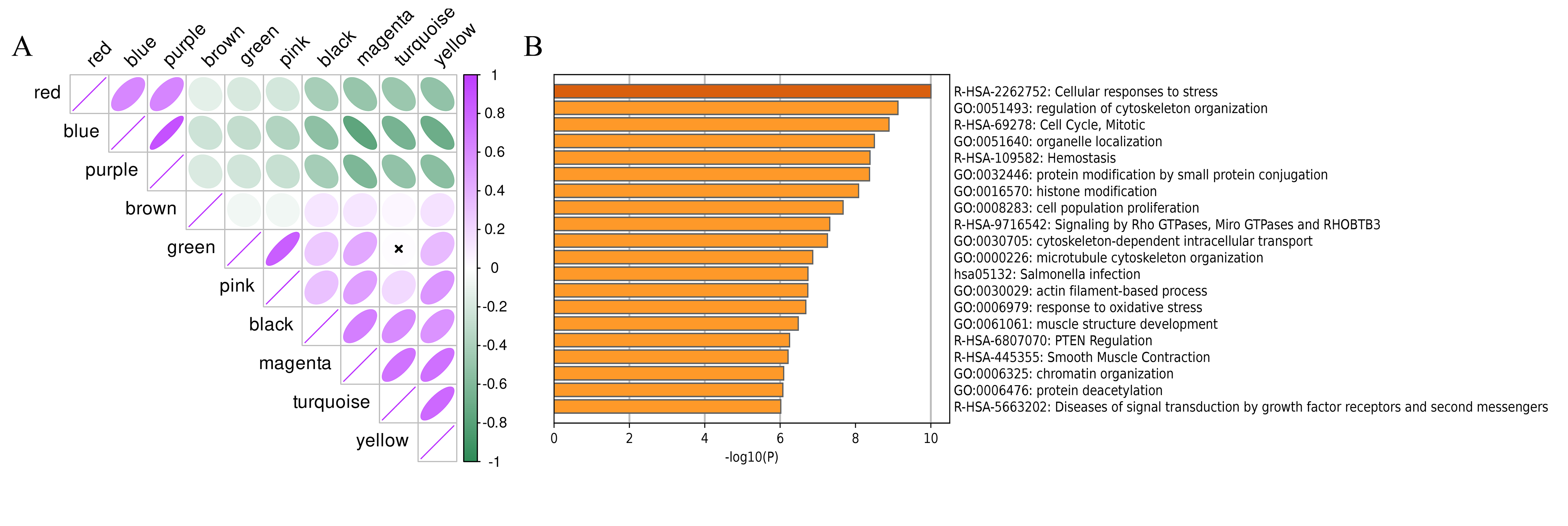


Figure S2: (A) Correlation analysis of the modules identified by hdWGCNA. (B) Enrichment analysis for the turquoise module.


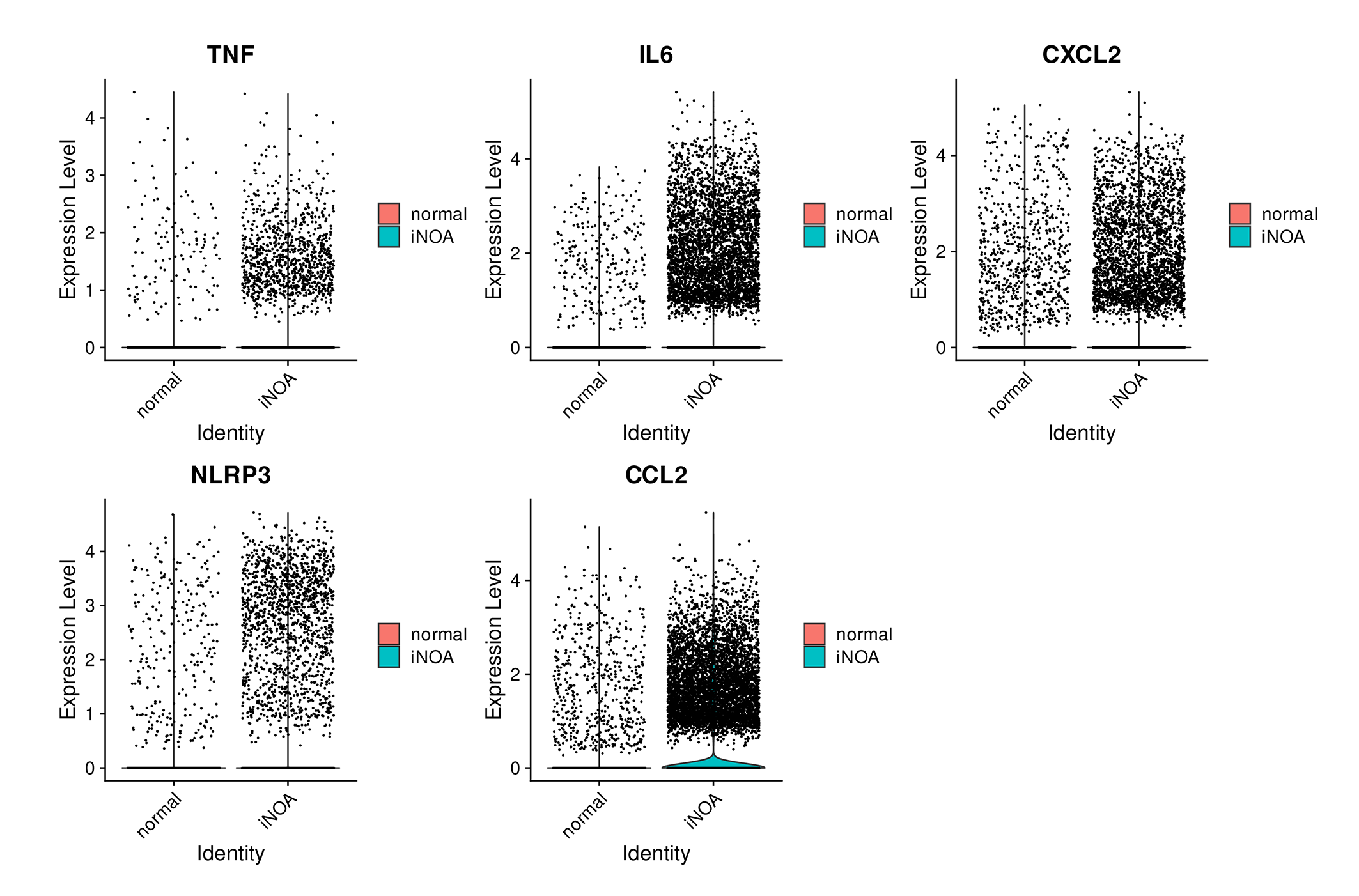


Figure S3: Violin plot for testicular inflammation genes.
